# Counselling treatment versus counselling associated with jaw exercises in patients with disc displacement with reduction—a single-blinded, randomized, controlled clinical trial

**DOI:** 10.1186/s12903-023-03096-7

**Published:** 2023-06-14

**Authors:** Carolina Antunes Santa Cecília Simões, Mayara Aparecida Moreira da Silva, Rafael Alvim Magesty, Saulo Gabriel Moreira Falci, Dhelfeson Willya Douglas-de-Oliveira, Patricia Furtado Gonçalves, Olga Dumont Flecha

**Affiliations:** Universidade Federal dos Vales do Jequitinhonha e Mucuri, Rua da Glória, 187, Centro, Diamantina, Minas Gerais 39100-000 Brazil

**Keywords:** Temporomandibular Disorders, Temporomandibular Joint Disc, Temporomandibular Joint Disorders / therapy, Temporomandibular Articular Disc

## Abstract

**Objective:**

To compare effectiveness of counselling program *versus* counselling program plus jaw exercises to reduce pain and click in patients with temporomandibular joint disc displacement with reduction (DDWR).

**Materials and methods:**

Patients were divided into two groups: instructions for temporomandibular disorders (TMD) plus jaw exercises (test, *n* = 34), only TMD instructions (control, *n* = 34). Pain was analyzed by palpation (RDC/TMD). It was investigated if the click caused discomfort. Both groups were evaluated at baseline, 24 h, 7 days, and 30 days’ post treatment.

**Results:**

The click was present in 85.7% (*n* = 60). In 30-day evaluation, there was a statistically significant difference between groups in the right median temporal muscle (*p* = 0.041); and there was a statistically significant difference in treatment self-perception (*p* = 0.002) and click’s discomfort (*p* < 0.001).

**Conclusion:**

The exercise with recommendations showed better results, resolution of the click, and self-perception of the treatment effectiveness.

**Clinical relevance:**

This study presents therapeutic approaches that are easy to perform and that can be monitored remotely. In view of the current stage of the global pandemic, these treatment options become even more valid and useful.

**Clinical trial register:**

This clinical trial was registered at Brazilian Clinical Trials Registry (ReBec) under protocol RBR-7t6ycp (http://www.ensaiosclinicos.gov.br/rg/RBR-7t6ycp/), Date of registration: 26/06/2020.

## Introduction

Temporomandibular disorders (TMD) are associated with some signs and symptoms such as tinnitus, earache, jaw locking, limitations and deviations in the mandibular trajectory, in addition to joint clicks during mouth opening or closing [1, 2]. According to the Diagnostic Criteria for TMD (DC/TMD) Axis I, TMD can be divided into myogenic disorders (Group I) and intra-capsular disorders, including disc displacements (Group II) or arthralgia, arthritis, and arthrosis (Group III) [1].

TMD is the second most common musculoskeletal disorder that causes pain and disability [3]. Although the therapy of temporomandibular joint disorders remains controversial [4], the first line TMD treatment is still considered a conservative approach, including physical therapy, oral nonsteroidal anti-inflammatory drugs (NSAIDs), laser therapy, transcutaneous electrical nerve stimulation (TENS), occlusal splints, extracorporeal shockwave therapy, and oxygen-ozone therapy [1].

In some cases, the click is usually caused by the anterior disc displacement with reduction, which can also be displaced to the posterior, medial, and lateral portion [2, 3]. This condition occurs both at the mouth opening and closing, and it can be painful or not [5–7]. Some factors can influence the disc displacement with reduction (DDWR), such as: micro or macro-trauma, lack of muscle coordination, joint hypermobility, severe injury or deviation in the condyle surface or in the temporomandibular joint (TMJ) fossa, decreased lubrification, TMJ flaccidity, among others [3, 5, 8, 9].

The prevalence of DDWR increases with age [10]. This may be related to the TMJ morphology and the dimensions of the joint space that change with aging, and the space decreasing can cause a disc dislocation [11, 12]. DDWR prevalence is higher in females, which may be related to a greater joint laxity [13, 14].

Clinically, DDWR can manifest in three patterns: the first is painless and discovered during a routine physical examination; the second is also painless but the patient seeks care to reduce or eliminate the click; and the third, when the click appears along with pain, is treated focusing on eliminating the arthralgia [15].

Some therapies, such as physiotherapy, pharmacotherapy, occlusal devices, and psychological therapy are used to treat DDWR. Arthrocentesis and viscosupplementation are also used as therapies for DDWR and can be performed alone or together [16, 17]. Non-invasive treatments, such as counselling for behavioral changes, are generally chosen as the first approach [1, 4]. In addition, jaw exercises can be effective for most patients with TMD-related pain [18]. Both above mentioned therapies have a high acceptance rate by the patients and are easily executed at home [18].

Thus, the aim of this study was to evaluate and compare the effectiveness for reducing pain and click perception in patients who received counseling only with those who received counseling associated with jaw exercises. Furthermore, the secondary objective was to assess the presence of parafunctional habits.

## Material and methods

### Study design

This study is a randomized single-blind clinical trial composed of two groups of 34 patients each, and it was conducted following the Consolidated Standards of Reporting Trials (CONSORT) [19]. This clinical trial was registered at Brazilian Clinical Trials Registry (http://www.ensaiosclinicos.gov.br/rg/RBR-7t6ycp/) on 26/06/2020. The follow-up period was 30 days. This study was approved by the Research Ethics Committee of the Universidade Federal dos Vales do Jequitinhonha e Mucuri (UFVJM), protocol #1.843.016, and conducted in accordance with the Helsinki declaration, revised in 2013.

Patients seeking for treatment at the Surgery and Periodontics Clinic of the UFVJM, with diagnosis of TMD, were recruited for the study. These patients were examined by a professional and those who met the inclusion criteria were invited to participate in the study, after signing a consent form. Those excluded patients were referred to appropriate treatment.

The inclusion criteria were patients aged 18 years or older, complaining of TMJ click (audible) during mouth opening/closing, followed or not by pain in the facial region, who never underwent any type of treatment for TMD’s. Exclusion criteria were: edentulous patients, patients who have already undergone any treatment for TMD’s; those patients with any systemic disease that could cause joint and/or muscle changes; and patients who were undergoing drug treatment for the condition.

### Study settings

This study was carried out at the Surgery and Periodontics Clinic of the UFVJM. A researcher (MAMS), properly calibrated and trained according to the video available on the RDC/TMD website (https://ubwp.buffalo.edu/rdc-tmdinternational), was responsible for carrying out all evaluations. Each patient was evaluated in four appointments: baseline, 24 h, seven and 30 days after the interventions. Another researcher was responsible for teaching the participants the jaw exercises and for giving the instructions (counselling).

### Questionnaire

After the patients’ admissions to the study, a specific clinical form for TMD, the Research diagnostic criteria for TMDs (RDC/TMD) [20], was filled out. At this point, the presence of TMJ clicks was investigated through clinical examination. Pain was analyzed by palpation, and measured based on the RDC/TMD. The patients were asked if the click was causing discomfort. The Oral Health Impact Profile (OHIP-14) questionnaire was also applied [21].

### Randomizing process and blinding

The randomization process was previously conducted by an independent researcher who was blinded to the patients and interventions. Each intervention was written, drawn, and sealed in opaque envelopes before starting the study. The interventions were drawn as “A” for the test group, which received jaw exercises and counselling, and “B” for the control group, which received only counselling program. Only the researcher responsible for the interventions knew the envelope content. The numbers corresponding to the patients and the interventions (classified as “A” and “B”) were placed in opaque envelopes. Thus, each patient had their corresponding numbers placed on the envelopes along with the intervention, assigned by lottery. Each patient received the allocated intervention, which was taken from the envelope at the appointment time, and the clinician only knew the intervention to be applied at the time to execute it. The allocation remained hidden until the time of the intervention. The interventions were always executed by the same researcher, who did not participate in the evaluations.

### Interventions

The patients were divided into two groups: the test group received a counselling program (Table 1) [22] plus jaw exercises (Table 2) [23], and the control group received only counselling. All exercises were instructed to be performed in a total of 20 repetitions, thrice a day, with no pre-established time restriction for each repetition, every day until the end of the research. The instructions for the counselling program and jaw exercises were read and explained in a standardized way by the same researcher. In addition, the patients received all written instructions.

**Table 1:** Counselling Program

Diet changing, limited to soft foods
Avoid habits that overload the masticatory muscles, such as chewing gum; also, avoiding yawning, yelling, singing, and long sessions at the dentist
Application of hot compress over the painful area for 20 min. Cold compress over the painful area for 10 min until feeling "tingling". This compresses combination should be done two to four times a day
Keep the teeth separated
Maintain good posture
Trying to improve sleep quality
Avoid parafunctional habits like biting nails and pencils
Decrease caffeine intake

**Table 2:** Jaw Exercises

Opening and closing the mouth slowly, with the vertex of the tongue positioned on the lingual face of the upper incisors
Pronouncing the letter "N" and keeping the tongue positioned on the lingual face of the upper incisors
Opening and closing the mouth slowly in front of a mirror with a straight vertical line drawn, trying to keep the midline of the smallest dental arch parallel to the mirror while performing the movement
Placing the chin in a closed hand during the jaw depression movement (mouth opening), and making it difficult to raise the mandible (mouth closing) by pressing the lower incisors with the index and middle finger; and pressing the lateral area of the mandible body with the index and middle fingers, exerting a force opposite to the movement performed

### Outcomes

The RDC/TMD questionnaire for muscle and joint palpation at specific points, which uses a pain scale from zero to three (0: pressure only; 1: mild pain; 2: moderate pain; 3: severe pain), was applied in the baseline and 24 h, seven and 30 days after the interventions. At each evaluation, the patients were asked if the pain level was “better”, “worse” or “the same” in relation to the previous evaluation, and if the click was still making them uncomfortable.

### Sample size

The sample size calculation was based on the following parameters: the standard deviation obtained from pain intensity through the visual analogic scale (13.1 mm) [18]; the difference stipulated between groups, which was 9 mm; a significance level of 95%; and a power of 80%. Thus, it was required 34 participants per group in the study.

### Statistical methods

**S**tatistical analyzes were performed using the statistical package SPSS® (Statistical Package for the Social Sciences Inc.) version 23.0. Exploratory analyzes provided frequencies, means and standard deviations. 95% confidence interval was used, and 5% level of significance was adopted. The data normal distribution was verified through the Shapiro–Wilk test. Chi-square test was used to verify the association between categorical variables. The inter- and intra-group comparisons were performed using the Mann–Whitney and Friedman tests (post hoc Wilcoxon) respectively.

## Results

**Fig. 1:**
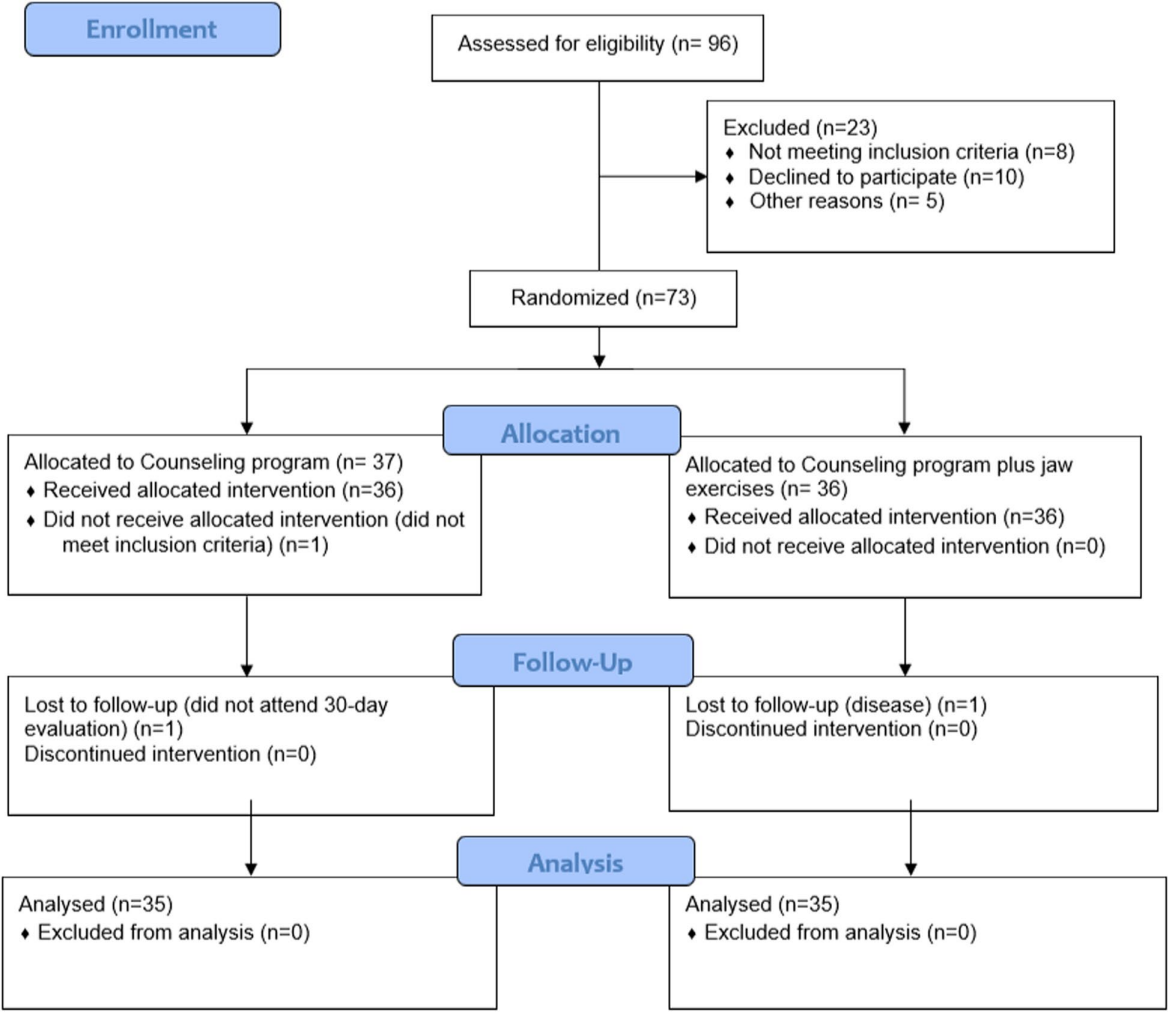
Flowchart of the included participants

The number of participants and the losses with the reasons are described in the Flowchart (Fig. 1). The prevalence of females (*n* = 48, 68.4%) was higher in the sample. The mean age was 25.88 (± 7.26) years.

The click during mouth opening was present in 60 patients (85.7%), while the click during mouth closing was observed in 29 patients (41.4%). Pain during maximum mouth opening (*n* = 30, 42.9%), mandibular protrusion (*n* = 16, 22.9%), and right and left laterality (*n* = 13, 18.6%) movements was present in the minority of patients.

Some types of habits were present: gnashing teeth (*n* = 25, 35.7%), biting nails (*n* = 19, 27.1%), chewing gum (*n* = 20, 28.6%), and using the phone (*n* = 21, 30%). Other habits, such as clenching teeth (*n* = 46, 65.7%), talking a lot (*n* = 42, 60%), stress (*n* = 40, 57.1%), and using computers (*n* = 46, 65.7%) were present in most of the participants.

In the baseline, patients of the counselling plus exercises group showed higher mean pain in the right side compared with the left side in five palpation points: medium temporal muscle (*p* = 0.027), anterior temporal muscle (*p* = 0.006), superior masseter muscle (*p* = 0.030), posterior mandibular (*p* = 0.023), and posterior ligament (*p* = 0.026) (Table 3).

**Table 3:** Palpation – Inter-groups comparisons

	**Baseline – Right side**					**Baseline – Left side**					**24-h evaluation – Right side**					**24-h evaluation – left side**				
	**Counselling**		**Counselling plus exercises**			**Counselling**		**Counselling plus exercises**			**Counselling**		**Counselling plus exercises**			**Counselling**		**Counselling plus exercises**		
	**Mean**	**SD**	**Mean**	**SD**	***p***	**Mean**	**SD**	**Mean**	**SD**	***p***	**Mean**	**SD**	**Mean**	**SD**	***p***	**Mean**	**SD**	**Mean**	**SD**	***p***
Posterior Temporal	0.03	0.171	0.06	0.236	0.575	0.03	0.171	0.14	0.494	0.307	0.03	0.17	0.00	0.00	0.317	0.03	0.17	0.03	0.17	0.999
Medium Temporal	0.24	0.606	0.17	0.453	0.883	0.09	0.379	0.37	0.731	**0.027**	0.18	0.46	0.09	0.29	0.434	0.06	0.24	0.09	0.29	0.644
Anterior Temporal	0.12	0.327	0.17	0.568	0.999	0.00	0.000	0.26	0.561	**0.006**	0.03	0.17	0.06	0.24	0.558	0.00	0.00	0.15	0.44	**0.041**
Superior Masseter	0.21	0.538	0.34	0.765	0.518	0.06	0.343	0.34	0.765	**0.030**	0.18	0.53	0.12	0.42	0.674	0.09	0.38	0.09	0.38	0.999
Medium Masseter	0.32	0.768	0.37	0.770	0.783	0.18	0.576	0.23	0.646	0.743	0.18	0.58	0.15	0.44	0.991	0.06	0.24	0.03	0.17	0.558
Inferior Masseter	0.21	0.641	0.37	0.877	0.486	0.12	0.537	0.31	0.718	0.090	0.15	0.44	0.09	0.38	0.410	0.03	0.17	0.03	0.17	0.999
Posterior Mandibular	0.09	0.514	0.29	0.750	0.108	0.00	0.000	0.29	0.750	**0.023**	0.09	0.52	0.09	0.38	0.582	0.00	0.00	0.06	0.35	0.317
Submandibular	0.09	0.514	0.14	0.494	0.434	0.12	0.478	0.20	0.632	0.432	0.12	0.55	0.03	0.17	0.546	0.09	0.38	0.03	0.17	0.546
Lateral Pole (TMJ)	0.38	0.817	0.51	0.781	0.266	0.26	0.666	0.51	0.887	0.174	0.21	0.65	0.21	0.48	0.557	0.12	0.42	0.27	0.67	0.282
Posterior Ligament (TMJ)	0.12	0.537	0.29	0.710	0.154	0.03	0.171	0.29	0.622	**0.026**	0.09	0.52	0.12	0.42	0.329	0.00	0.00	0.09	0.38	0.154
Lateral Pterygoid	0.18	0.626	0.37	0.731	0.082	0.12	0.537	0.20	0.632	0.425	0.12	0.42	0.18	0.58	0.692	0.00	0.00	0.12	0.55	0.154
Tendon of the temporal	0.29	0.799	0.29	0.710	0.828	0.21	0.641	0.43	0.815	0.146	0.21	0.65	0.27	0.67	0.524	0.09	0.38	0.33	0.78	0.127
	**7-day – Right side**					**7-day – Left side**					**30-day evaluation – Right side**					**30-day evaluation – left side**				
	**Counselling**		**Counselling plus exercises**			**Counselling**		**Counselling plus exercises**			**Counselling**		**Counselling plus exercises**			**Counselling**		**Counselling plus exercises**		
	**Mean**	**SD**	**Mean**	**SD**	**p**	**Mean**	**SD**	**Mean**	**SD**	**p**	**Mean**	**SD**	**Mean**	**SD**	**p**	**Mean**	**SD**	**Mean**	**SD**	**p**
Posterior Temporal	0.00	0.00	0.03	0.17	0.317	0.00	0.00	0.00	0.00	0.999	0.00	0.00	0.00	0.00	0.999	0.03	0.17	0.00	0.00	0.317
Medium Temporal	0.09	0.38	0.09	0.29	0.675	0.09	0.29	0.03	0.17	0.306	0.12	0.33	0.00	0.00	0**.041**	0.09	0.29	0.03	0.17	0.306
Anterior Temporal	0.03	0.17	0.12	0.42	0.299	0.06	0.24	0.03	0.17	0.558	0.03	0.17	0.06	0.35	0.983	0.03	0.17	0.09	0.38	0.546
Superior Masseter	0.09	0.38	0.13	0.42	0.629	0.00	0.00	0.09	0.38	0.154	0.06	0.35	0.03	0.17	0.983	0.00	0.00	0.03	0.17	0.317
Medium Masseter	0.09	0.29	0.12	0.33	0.692	0.06	0.24	0.00	0.00	0.154	0.06	0.24	0.03	0.17	0.558	0.03	0.17	0.03	0.17	0.999
Inferior Masseter	0.12	0.42	0.09	0.29	0.969	0.03	0.17	0.03	0.17	0.999	0.06	0.24	0.03	0.17	0.558	0.09	0.29	0.03	0.17	0.306
Posterior Mandibular	0.09	0.52	0.09	0.29	0.329	0.00	0.00	0.03	0.17	0.317	0.06	0.24	0.03	0.17	0.558	0.03	0.17	0.00	0.00	0.317
Submandibular	0.06	0.35	0.09	0.38	0.570	0.00	0.00	0.00	0.00	0.999	0.00	0.00	0.00	0.00	0.999	0.03	0.17	0.03	0.17	0.999
Lateral Pole (TMJ)	0.15	0.62	0.03	0.17	0.534	0.06	0.35	0.03	0.17	0.983	0.15	0.44	0.09	0.38	0.410	0.09	0.38	0.15	0.62	0.963
Posterior Ligament (TMJ)	0.12	0.55	0.03	0.17	0.546	0.06	0.35	0.00	0.00	0.317	0.12	0.55	0.09	0.52	0.570	0.09	0.29	0.00	0.00	0.079
Lateral Pterygoid	0.15	0.62	0.09	0.38	0.963	0.00	0.00	0.06	0.35	0.317	0.12	0.42	0.09	0.29	0.969	0.12	0.42	0.06	0.35	0.321
Tendon of the temporal	0.15	0.62	0.09	0.38	0.963	0.00	0.00	0.06	0.35	0.317	0.09	0.29	0.03	0.17	0.306	0.03	0.17	0.03	0.17	0.999

^Mann–Whitney test^

In the 24-h evaluation, the left side of the anterior temporal muscle showed higher mean of pain on palpation in the inter group analysis (*p* = 0.041) (Table 3).

No statistically significant difference was found at any point in the seven-day assessment (Table 3). In the 30-day evaluation, the right medium temporal muscle showed higher mean pain in the inter group analysis (Table 3).

In the counselling group, there was a significant intra-group difference in the lateral pole (TMJ) on the right side (*p* = 0.016) (Table 4) and on the left side (*p* = 0.033) evaluation (Table 4).

**Table 4:** Palpation – Intra-group comparison

	**Counselling – Right side**										**Counselling – Left side**									
	**Baseline (T1)**		**24-h (T2)**		**7 days (T3)**		**30 days (T4)**				**Baseline (T1)**		**24-h (T2)**		**7 days (T3)**		**30 days (T4)**			
	**Mean**	**SD**	**Mean**	**SD**	**Mean**	**SD**	**Mean**	**SD**	***p***	**post hoc**	**Mean**	**SD**	**Mean**	**SD**	**Mean**	**SD**	**Mean**	**SD**	***p***	**post hoc**
Posterior Temporal	0.03	0.17	0.03	0.17	0.00	0.00	0.00	0.00	0.392		0.03	0.17	0.03	0.17	0.00	0.00	0.03	0.17	0.733	
Medium Temporal	0.24	0.61	0.18	0.46	0.09	0.38	0.12	0.33	0.481		0.09	0.38	0.06	0.24	0.09	0.29	0.09	0.29	0.910	
Anterior Temporal	0.12	0.33	0.03	0.17	0.03	0.17	0.03	0.17	0.145		0.00	0.00	0.00	0.00	0.06	0.24	0.03	0.17	0.300	
Superior Masseter	0.21	0.54	0.18	0.53	0.09	0.38	0.06	0.35	0.083		0.06	0.34	0.09	0.38	0.00	0.00	0.00	0.00	0.300	
Medium Masseter	0.32	0.77	0.18	0.58	0.09	0.29	0.06	0.24	0.115		0.18	0.58	0.06	0.24	0.06	0.24	0.03	0.17	0.096	
Inferior Masseter	0.21	0.64	0.15	0.44	0.12	0.42	0.06	0.24	0.532		0.12	0.54	0.03	0.17	0.03	0.17	0.09	0.29	0.300	
Posterior Mandibular	0.09	0.51	0.09	0.52	0.09	0.52	0.06	0.24	0.801		0.00	0.00	0.00	0.00	0.00	0.00	0.03	0.17	0.392	
Submandibular	0.09	0.51	0.12	0.55	0.06	0.35	0.00	0.00	0.204		0.12	0.48	0.09	0.38	0.00	0.00	0.03	0.17	0.468	
Lateral Pole (TMJ)	0.38	0.82	0.21	0.65	0.15	0.62	0.15	0.44	**0.016**	T1xT2: 0.059 T1xT3: 0.020 T1xT4: 0.035 T2xT3: 0.157 T2xT4: 0.317 T3xT4: 0.999	0.26	0.67	0.12	0.42	0.06	0.35	0.09	0.38	**0.033**	T1xT2: 0.102 T1xT3: 0.034 T1xT4: 0.161 T2xT3: 0.157 T2xT4: 0.705 T3xT4: 0.655
Posterior Ligament (TMJ)	0.12	0.54	0.09	0.52	0.12	0.55	0.12	0.55	0.801		0.03	0.17	0.00	0.00	0.06	0.35	0.09	0.29	0.284	
Lateral Pterygoid	0.18	0.63	0.12	0.42	0.15	0.62	0.12	0.42	0.910		0.12	0.54	0.00	0.00	0.00	0.00	0.12	0.42	0.112	
Tendon of the temporal	0.29	0.80	0.21	0.65	0.15	0.62	0.09	0.29	0.446		0.21	0.64	0.09	0.38	0.00	0.00	0.03	0.17	0.232	
	**Counselling plus exercises – Right side**										**Counselling plus exercises – Left side**									
	**Baseline (T1)**		**24-h (T2)**		**7 days (T3)**		**30 days (T4)**				**Baseline (T1)**		**24-h (T2)**		**7 days (T3)**		**30 days (T4)**			
	**Mean**	**SD**	**Mean**	**SD**	**Mean**	**SD**	**Mean**	**SD**	***p***	**post hoc**	**Mean**	**SD**	**Mean**	**SD**	**Mean**	**SD**	**Mean**	**SD**	***p***	**post hoc**
Posterior Temporal	0.06	0.24	0.00	0.00	0.03	0.17	0.00	0.00	0.300		0.14	0.49	0.03	0.17	0.00	0.00	0.00	0.00	0.145	
Medium Temporal	0.17	0.45	0.09	0.29	0.09	0.29	0.00	0.00	0.084		0.37	0.73	0.09	0.29	0.03	0.17	0.03	0.17	**0.002**	T1xT2: 0.031 T1xT3: 0.010 T1xT4: 0.013 T2xT3: 0.157 T2xT4: 0.317 T3xT4: 0.999
Anterior Temporal	0.17	0.57	0.06	0.24	0.12	0.42	0.06	0.35	0.436		0.26	0.56	0.15	0.44	0.03	0.17	0.09	0.38	**0.049**	T1xT2: 0.157 T1xT3: 0.023 T1xT4: 0.177 T2xT3: 0.102 T2xT4: 0.589 T3xT4: 0.414
Superior Masseter	0.34	0.76	0.12	0.42	0.13	0.42	0.03	0.17	**0.049**	T1xT2: 0.071 T1xT3:0.084 T1xT4: 0.026 T2xT3: 0.999 T2xT4: 0.257 T3xT4: 0.257	0.34	0.76	0.09	0.38	0.09	0.38	0.03	0.17	**0.024**	T1xT2: 0.041 T1xT3: 0.101 T1xT4: 0.027 T2xT3: 0.999 T2xT4: 0.414 T3xT4: 0.414
Medium Masseter	0.37	0.77	0.15	0.44	0.12	0.33	0.03	0.17	**0.004**	T1xT2: 0.054 T1xT3: 0.046 T1xT4: 0.010 T2xT3: 0.546 T2xT4: 0.046 T3xT4: 0.083	0.23	0.65	0.03	0.17	0.00	0.00	0.03	0.17	**0.025**	T1xT2: 0.301 T1xT3: 0.039 T1xT4: 0.084 T2xT3: 0.317 T2xT4: 0.999 T3xT4: 0.317
Inferior Masseter	0.37	0.88	0.09	0.38	0.09	0.29	0.03	0.17	0.100		0.31	0.72	0.03	0.17	0.03	0.17	0.03	0.17	**0.004**	T1xT2: 0.026 T1xT3: 0.026 T1xT4: 0.031 T2xT3: 0.999 T2xT4: 0.999 T3xT4: 0.999
Posterior Mandibular	0.29	0.75	0.09	0.38	0.09	0.29	0.03	0.17	0.084		0.29	0.75	0.06	0.35	0.03	0.17	0.00	0.00	**0.006**	T1xT2: 0.066 T1xT3: 0.041 T1xT4: 0.039 T2xT3: 0.317 T2xT4: 0.317 T3xT4: 0.317
Submandibular	0.14	0.49	0.03	0.17	0.09	0.38	0.00	0.00	0.290		0.20	0.63	0.03	0.17	0.00	0.00	0.03	0.17	0.080	
Lateral Pole (TMJ)	0.51	0.78	0.21	0.48	0.03	0.17	0.09	0.38	** < 0.001**	T1xT2: 0.020 T1xT3: 0.002 T1xT4: 0.003 T2xT3: 0.034 T2xT4: 0.305 T3xT4: 0.414	0.51	0.89	0.27	0.67	0.03	0.17	0.15	0.62	**0.001**	T1xT2: 0.041 T1xT3: 0.004 T1xT4: 0.051 T2xT3: 0.039 T2xT4: 0.389 T3xT4: 0.180
Posterior Ligament (TMJ)	0.29	0.71	0.12	0.42	0.03	0.17	0.09	0.52	0.059		0.29	0.62	0.09	0.38	0.00	0.00	0.00	0.00	**0.001**	T1xT2: 0.294 T1xT3: 0.412 T1xT4: 0.412 T2xT3: 0.118 T2xT4: 0.118 T3xT4: < 0.001
Lateral Pterygoid	0.37	0.73	0.18	0.58	0.09	0.38	0.09	0.29	**0.020**	T1xT2: 0.053 T1xT3: 0.013 T1xT4: 0.039 T2xT3: 0.083 T2xT4: 0.527 T3xT4: 0.999	0.20	0.63	0.12	0.55	0.06	0.35	0.06	0.35	0.125	
Tendon of the temporal	0.29	0.71	0.27	0.67	0.09	0.38	0.03	0.17	**0.021**	T1xT2: 0.564 T1xT3: 0.053 T1xT4: 0.047 T2xT3: 0.034 T2xT4: 0.054 T3xT4: 0.414	0.43	0.81	0.33	0.78	0.06	0.35	0.03	0.17	**0.001**	T1xT2: 0.157 T1xT3: 0.006 T1xT4: 0.011 T2xT3: 0.024 T2xT4: 0.040 T3xT4: 0.655

Friedman test

post hoc Wilcoxon (*p*<0.05)

In the intra-group analysis, the right side showed higher mean of pain on palpation in five muscles, in the counselling plus exercises group: upper masseter muscle (*p* = 0.049), medium masseter (*p* = 0.004), lateral pole (TMJ) (*p* < 0.001), lateral pterygoid muscle (*p* = 0.02), and temporal muscle tendon (*p* = 0.021) (Table 4). On the left side of this group, it was observed that two palpation sites did not show statistically significant results: submandibular (*p* = 0.08) and lateral pterygoid (*p* = 0.125) (Table 4).

A statistically significant difference in the intergroup analysis was found in the self-perception of the treatment result (*p* = 0.002) and in the click discomfort (*p* < 0.001), after 30 days of intervention (Table 5). In the intra-group comparison, a statistically significant difference was found in the self-perception of the treatment result (*p* < 0.001) and in the discomfort (*p* < 0.001), when evaluating the counselling plus jaw exercises group (Table 6). Outcomes collected from patient reports were published in another study [20].

**Table 5:** Questionnaire – Inter-groups comparison

		Counselling		Counselling plus exercises		
		*n*	%	*n*	%	*p*
**24-h**	How do you consider your treatment result?					
	Better	7	21.2	6	18.2	
	Same	26	78.8	25	75.8	
	Worse	0	0.0	2	6.1	0.659
	Does the TMJ click still bother you?					
	No	10	30.3	11	33.3	
	Yes	23	69.7	22	66.7	0.766
**7 days**	How do you consider your treatment result?					
	Better	12	36.4	21	63.6	
	Same	18	54.5	12	36.4	
	Worse	3	9.1	0	0.0	0.105
	Does the TMJ click still bother you?					
	No	9	27.3	17	51.5	
	Yes	24	72.7	16	48.5	0.094
**30 days**	How do you consider your treatment result?					
	Better	12	36.4	28	84.8	
	Same	19	57.6	5	15.2	
	Worse	2	6.1	0	0.0	**0.002**
	Does the TMJ click still bother you?					
	No	6	18.2	29	87.9	
	Yes	27	81.8	4	12.1	** < 0.001**

Chi−Square test

**Table 6:** Questionnaire – Intra-group comparison

	Counselling plus exercises						
	24-h		7 days		30 days		
	*n*	%	*n*	%	*n*	%	*p*
How do you consider your treatment result?							
Better	6	18.2	21	63.6	28	84.8	** < 0.001**
Same	25	75.8	12	36.4	5	15.2	
Worse	2	6.1	0	0.0	0	0.0	
Does the TMJ click still bother you?							
No	11	33.3	17	51.5	29	87.9	** < 0.001**
Yes	22	66.7	16	48.5	4	12.1	

^Chi-Square test^

## Discussion

Both the counselling group and the counselling plus jaw exercises group showed effectiveness in the TMD treatment, improving the patients’ symptoms when comparing the follow-up evaluations to the baseline. This study reported a statistically significant difference for the resolution of the TMJ click at the end of the follow-up period when comparing the two treatment groups, in which the counselling plus jaw exercises group showed better results.

Regarding the palpation, the counselling plus jaw exercises therapy showed higher efficacy throughout the evaluations when compared to the control group. This may be related to the fact that the exercises contribute to an improvement in the biomechanical activity of the articular disc and help reestablishing the muscular function [24].

Palpation on the condyle lateral poles showed statistically significant results at all evaluations in both comparison groups. This may suggest that treatment including only the counselling program is effective to treat DDWR. Corroborating this fact, a systematic review [25] reported that counselling was able to improve the sensitivity to muscle palpation and the maximum mouth opening. In addition, another study demonstrated that only counselling was effective to treat TMDs, resulting in significant improvements in pain and quality of life [26].

In the 30-day evaluation, the patients who received the counselling plus jaw exercises were unanimous when reporting that the treatment did not worsen the condition, unlike the patients who received only counselling. This may be linked to the fact that the exercises included in the present study protocol are efficient for the DDWR [7], since another study [27] reported that the exercises of their protocol were seen as a limitation. Thus, in that study, there was no statistically significant difference between the comparison groups (with and without the exercises) in a follow-up period of four to six weeks [27].

The TMJ click is one of the most common complaints among TMD patients, what was also verified in this study [28]. After 30 days of follow-up, most of the patients in the counselling plus exercises group showed that the click was not bothering them anymore, unlike the group treated with counselling only. This reinforces the effectiveness of the jaw exercises also reported in other studies [7, 15, 23, 29, 30].

The sample was composed mainly of female patients, who are more likely to develop DDWR because they have a looser joint and, when aging, this tends to worsen since the disc position usually changes [10, 13, 14]. Among the parafunctional habits reported by the patients, it is important to highlight the “stress” and “clenching teeth”, which are directly connected to each other, and can lead to the development of TMDs or even aggravate the condition already present [31, 32].

To reduce the risk of bias and to present a high level of evidence and internal validity, this study followed the CONSORT checklist for a clinical trial, including randomization, allocation concealment, blinding, sample size calculation, and analysis by protocol [33].

### Limitations

There are limitations to be considered in this study: the absence of a group treated only with exercises and a control group without any treatment. The latter would probably not be approved by the University Research Ethics Committee due to the presence of patients with pain. Therefore, it was decided for a group with orientations/ counselling.

The follow-up period may also be seen as a limitation, as some studies suggest a follow-up of more than 30 days to assess the therapies effectiveness [18, 26]. However, this randomized clinical trial showed significant results within 30-days.

Regarding pain, it is also important to consider that the clinical improvement of the DDWR can be attributed to the cyclical aspect of the dysfunction itself [34], or even an improvement due to the simple fact that the patient was included in a clinical trial, phenomenon known as the Hawthorne effect [35]. In other patients, the pain may have a reason other than DDWR.

### Clinical implication

This clinical trial has a high external validity because it evaluates therapeutic approaches easy to conduct and can be taught and monitored remotely [18, 36]. In the current global pandemic stage, with the restrictions of human proximity and mobility imposed by the consequences of the Sars-CoV-2, these treatment options become even more valid and useful [37]. Moreover, the low number of clinical trials comparing the two therapeutic approaches for DDWR, with high methodological rigor, make this study relevant [38]. The treatment evaluated herein demonstrated to be efficient, easy to understand, low cost, and it can be used by clinicians as a viable alternative therapy. Future research on this subject must focus on negative control group comparison and a long follow-up [39].

## Conclusion

There was no difference between the two treatments in the improvement of the pain level. Patients that received counselling plus exercises showed greater results regarding the palpation points in the intra-group comparisons, TMJ click resolution, and self-perception of the treatment effectiveness when compared to the patients that received only counselling.

## Data Availability

The datasets used and/or analysed during the current study are available from the corresponding author on reasonable request.

## Abbreviations

CONSORT: Consolidated standards of reporting trials
DDWR: Disc displacement with reduction
OHIP-14: Oral health impact profile
RDC: Research diagnostic criteria
SPSS: Statistical package for the social sciences
TMD: Temporomandibular disorders
TMJ: Temporomandibular joint
UFVJM: Universidade Federal dos Vales do Jequitinhonha e Mucuri
